# Impact of Cardiovascular Failure in Intensive Care Unit-Acquired Pneumonia: A Single-Center, Prospective Study

**DOI:** 10.3390/antibiotics10070798

**Published:** 2021-06-30

**Authors:** Ignacio Martin-Loeches, Adrian Ceccato, Marco Carbonara, Gianluigi li Bassi, Pierluigi di Natale, Stefano Nogas, Otavio Ranzani, Carla Speziale, Tarek Senussi, Francesco Idone, Anna Motos, Miquel Ferrer, Antoni Torres

**Affiliations:** 1 Hospital Clinic, IDIBAPS, Universidad de Barcelona, CIBERes, 08036 Barcelona, Spain; drmartinloeches@gmail.com (I.M.-L.); adrianceccato@gmail.com (A.C.); marco.carbonara@gmail.com (M.C.); g.libassi@uq.edu.au (G.l.B.); digiannatale@clinic.cat (P.d.N.); stefano.nogas@gmail.com (S.N.); otavioranzani@yahoo.com.br (O.R.); carla.speziale11@gmail.com (C.S.); t.senussi@hotmail.it (T.S.); axl38@hotmail.com (F.I.); amotos@clinic.cat (A.M.); miferrer@clinic.cat (M.F.); 2 Department of Intensive Care Medicine, St. James’s University Hospital, Multidisciplinary Intensive Care Research Organization (MICRO), Trinity Centre for Health Sciences, D08 NHY1 Dublin, Ireland

**Keywords:** VAP, pneumonia, shock, antibiotics, ICUAP

## Abstract

Background: Cardiovascular failure (CVF) may complicate intensive care unit-acquired pneumonia (ICUAP) and radically alters the empirical treatment of this condition. The aim of this study was to determine the impact of CVF on outcome in patients with ICUAP. Methods: A prospective, single-center, observational study was conducted in six medical and surgical ICUs at a University Hospital. CVS was defined as a score of 3 or more on the cardiovascular component of the Sequential Organ Failure Assessment (SOFA) score. At the onset of ICUAP, CVF was reported as absent, transient (if lasting ≤ 3 days) or persistent (>3 days). The primary outcome was 90-day mortality modelled through a Cox regression analysis. Secondary outcomes were 28-day mortality, hospital mortality, ICU length of stay (LOS) and hospital LOS. Results: 358 patients were enrolled: 203 (57%) without CVF, 82 (23%) with transient CVF, and 73 (20%) with persistent CVF. Patients with transient and persistent CVF were more severely ill and presented higher inflammatory response than those without CVF. Despite having similar severity and aetiology, the persistent CVF group more frequently received inadequate initial antibiotic treatment and presented more treatment failures than the transient CVF group. In the persistent CVF group, at day 3, a bacterial superinfection was more frequently detected. The 90-day mortality was significantly higher in the persistent CVF group (62%). The 28-day mortality rates for patients without CVF, with transient and with persistent CVF were 19, 35 and 41% respectively and ICU mortality was 60, 38 and 19% respectively. In the multivariate analysis chronic pulmonary conditions, lack of Pa0_2_/FiO_2_ improvement at day 3, pulmonary superinfection at day 3 and persistent CVF were independently associated with 90-day mortality in ICUAP patients. *Conclusions*: Persistent CVF has a significant impact on the outcome of patients with ICUAP. Patients at risk from persistent CVF should be promptly recognized to optimize treatment and outcomes.

## 1. Introduction

Despite the success of several pre-emptive and therapeutic strategies in recent years [1,2,3], patients who develop cardiovascular failure (CVF) due to sepsis present high mortality rates, ranging between 20 and 40% depending on the population studied [4,5,6]. Almost a third of patients admitted to intensive care units (ICU) develop CVF during their stay [7,8]. CVF may complicate prognosis by adding secondary organ dysfunctions. It is defined as the need for vasopressor administration with persisting hypotension in order to maintain mean arterial pressure (MAP) ≥ 65 mm Hg. The lung is the most frequent source of infection in cases of CVF due to hospital-acquired infections [9].

ICU-acquired pneumonia (ICUAP) is the most common hospital-acquired infection in the ICU. This infection encompasses two different entities: ventilator-associated pneumonia (VAP) and hospital-acquired pneumonia (HAP) in non-intubated patients during their ICU stay [10,11]. Despite its high crude mortality, the overall attributable mortality of VAP is estimated at 13% [12].

An analysis of prognostic factors associated with HAP in the ICU showed that CVF was independently associated with a poor prognosis [13]. Nevertheless, few studies have assessed the impact of CVF in patients with HAP in the ICU [14,15,16]. Unfortunately, most of these studies are limited by the small sample size, inconsistency in the diagnostic criteria for pneumonia, the heterogeneity of the population studied, or the focus on VAP. We previously reported that around 40% cases of ICUAP occurred in patients without previous mechanical ventilation [17]. Therefore, the impact of CVF on ICUAP outcomes has still not been fully defined.

The aim of this study was to clinically evaluate patients with ICUAP who develop CVF; to assess their systemic inflammatory response; to identify risk factors for the development of CVF, and to evaluate the impact of CVF on outcomes such as mortality and ICU and hospital length of stay. In this manuscript, CVF is defined only as a natural consequence of septic shock and not as a “*primum movens*” pathological element.

## 2. Materials and Methods

### 2.1. Study Population

From January 2007 to August 2017 all patients older than 18 years admitted to six medical and surgical ICUs containing a total of 45 beds in an 800-bed university hospital, and with clinical suspicion of ICUAP, were prospectively and consecutively included in this study. Exclusion criteria were neutropenia after chemotherapy, hematopoietic transplant, drug-induced immunosuppression in solid-organ transplant, cytotoxic therapy, and clinical suspicion of aspiration pneumonia. The investigators made daily ICU rounds to recruit potential patients. Demographic, clinical and outcome data of ICUAP patients were prospectively collected.

The Institutional Review Board of Hospital Clinic, Barcelona (Spain) approved the study (registry number 2009/5427), and written informed consent was obtained from patients or their next-of-kin.

### 2.2. Data Collection

All relevant data from the medical records and bedside flow charts of patients, including demographic, clinical, laboratory, radiology, and microbiologic information, were collected at admission and at onset of pneumonia. Patients were followed up until hospital discharge or death.

### 2.3. Definition of Cardiovascular Failure

The Sequential (Sepsis-related) Organ Failure Assessment (SOFA) score was calculated in all patients upon ICU admission, and every day after inclusion in the study (at the time of ICUAP clinical diagnosis). CVF was defined as a score ≥ 3 on the cardiovascular component of the SOFA.

In view of previous observations, we established 72 h as the time to evaluate the response to treatment for CVF [18,19,20]. Therefore, we clustered patients in three groups: group A, no CVF during the course of ICUAP; group B, transient CVF, defined as presence of CVF at onset of ICUAP that resolved within three days; group C, persistent CVF, defined as the presence of CVF at onset of ICUAP that persisted for more than three days. Persistence of CVF was defined by a score ≥ 3 of the cardiovascular component of the SOFA score over 72 h.

### 2.4. Definition of Pneumonia, Microbiology Studies and Treatment

We defined ICUAP as pneumonia developing in patients admitted to the ICU for ≥48 h. The clinical suspicion of pneumonia was based on clinical criteria: new or progressive radiological pulmonary infiltrate together with at least two of the following: temperature > 38 °C or <36 °C, leucocytosis > 12,000/mm^3^ or leukopenia < 4000/mm^3^, and purulent respiratory secretion [21,22,23,24]. We considered VAP in patients with invasive mechanical ventilation for 48 h or more. Patients were classified as VAP or non-ventilator ICUAP, i.e., cases who did not meet VAP criteria [17].

Within 24 h of inclusion in the study [25], blood cultures and at least one lower respiratory sample were collected: i.e., sputum or airway secretions through a tracheobronchial aspirate, protected specimen brush, bronchoscopic [26] or blind bronchoalveolar lavage [27]. When clinically indicated, pleural fluids were cultured and, three days after inclusion, an additional respiratory sample was obtained. The presence in the second respiratory samples (day 3) of at least one new pathogen was defined as superinfection.

In case of suspicion of pneumonia caused by *Streptococcus pneumonia* or *Legionella pneumophila*, urinary antigen tests were performed. Microbiological confirmation of ICUAP was defined as the presence of at least one potentially pathogenic microorganism in pleural fluids, in respiratory samples above predefined thresholds (protected specimen brush ≥10^3^, bronchoalveolar lavage ≥ 10^4^, and sputum or tracheobronchial aspirates ≥10^5^ colony-forming units/mL) [28,29], or in blood cultures, assuming that any alternative cause of bacteraemia was ruled out [21,30].

Tests for microbial identification and antibiotic susceptibility were performed using standard methods [31]. Polymicrobial pneumonia was defined when more than one pathogenic microorganism was identified. The initial empirical antimicrobial treatment was administered according to the American Thoracic Society (ATS)/Infectious Disease Society of America (IDSA) guidelines [21] and adapted according to local ecology and the most common pattern of antimicrobial sensitivity. After 2–3 days, therapy was revised and adjusted according to the microbiology results. The empirical antimicrobial treatment was considered appropriate if the isolated pathogens were in vitro susceptible to at least one of the antimicrobials administered. In case of infections caused by *Pseudomonas aeruginosa*, an appropriate treatment required a combination of two active antibiotics against the isolated strain [32]. The initial response to treatment was evaluated after 72 h of antimicrobial treatment. Treatment failure was defined as at least one of the following criteria [19,33]: (1) no improvement of arterial oxygen tension to inspired oxygen fraction ratio, or intubation due to pneumonia (defined as the need for intubation 24 h after the beginning of antibiotic administration); (2) persistence of fever (>38 °C) or hypothermia (<35.5 °C) together with purulent respiratory secretions; (3) increase in the pulmonary infiltrates on chest radiograph of 50% or more; (4) occurrence of CVF (1) or multiple organ failure, defined as new dysfunctions of three or more organs [34]. In all patients who did not respond to treatment, respiratory samples and blood were cultured again, and the empirical antimicrobial treatment adjusted accordingly.

### 2.5. Data Analysis

Data are reported as means ± standard deviations or absolute numbers (percentage). Qualitative or categorical variables were compared with the chi-square or, when appropriate, Fisher’s exact test. Quantitative continuous variables were compared using the unpaired Student t test or the Mann-Whitney nonparametric test. Univariate logistic regression analysis for the clinically relevant variables was performed, and variables found with a significance level of *p* < 0.1 were included in the multivariate Cox regression model, whereas the excluded variables were highly correlated (r > |±0.3). The final selection of the variables associated with mortality was performed with subsequent stepwise forward method, for which the conditional criteria were used. A two-sided *p* value < 0.05 was considered statistically significant. All analyses were performed using SPSS software (Version 19, SPSS Inc., Chicago, IL, USA).

### 2.6. Primary and Secondary Outcomes

The primary outcome of this study was 90-day mortality in the three study groups (no CVF, transient CVF and persistent CVF). Secondary outcomes were ICU length of stay, ICU mortality, 28-day mortality and days of hospital stay.

## 3. Results

### 3.1. Study Sample

A total of 361 consecutive patients with ICUAP were prospectively enrolled, three of whom were excluded because the SOFA score was not calculated. Therefore, the study population consisted of 358 patients: 203 without CVF at onset of VAP, and 155 (43%) with CVF, transient in 82 (23%) and persistent in 73 (20%).

The characteristics of patients at admission and at onset of pneumonia are shown in Table 1 and Table 2. Patients with CVF had a higher proportion of chronic liver disease, a lower proportion of recent surgery, higher severity scores at ICU admission and at onset of pneumonia than patients without CVF; in addition, among the reasons for ICU admission, multiple trauma, lower body temperature were less frequent in patients with CVF, and multi-lobar and bilateral infiltrates and Acute Respiratory Distress Syndrome (ARDS) criteria were more frequent. Among patients with non-ventilator ICUAP, those with CVF required subsequent intubation more frequently than those without. None of these variables differed significantly between patients with transient and persistent CVF.

### 3.2. Characteristics upon ICUAP Diagnosis

Table 2 displays critical clinical data upon ICUAP diagnosis. Pneumonia was more severe in patients with CVF, as demonstrated by the need for orotracheal intubation, more extensive pulmonary infiltrations, and ARDS criteria. Again, all severity scores were higher in ICUAP patients who developed CVF. As shown in Table 2, several clinical parameters were associated with the onset of CVF in ICUAP patients; nevertheless, in the multivariate analysis, SOFA score at the time of pneumonia diagnosis, need for orotracheal intubation and C-reactive protein three days after ICUAP diagnosis were the only predictors of CVF.

### 3.3. Inflammatory Markers

Table 3 and Table 4 report data on inflammatory biomarkers at the time of pneumonia diagnosis and three days after ICUAP onset. Clearly, on diagnosis of the infection patients with CVF presented higher levels of PCT, IL-6, pro-adrenomedullin and suPAR, regardless of the duration; however, at day 3 after diagnosis, high biomarker levels were found only in patients with persistent CVF. At day 3, CRP also presented statistically significant differences between patients without CVF and those with persistent CVF.

### 3.4. Aetiology and Antibiotic Treatment

As Table 5 shows, there were no significant statistical differences regarding the causative pathogen of ICUAP between patients with or without CVF. However, patients with persistent CVF more frequently received inadequate antibiotic treatment likely to lead to treatment failure (this occurred in 78% of cases); furthermore, they did not present more frequent antibiotic changes. Causative pathogens of ICUAP are displayed in Table 6; no differences in specific pathogens were observed between patients with or without CVF.

### 3.5. Outcomes

**Figure 1:**
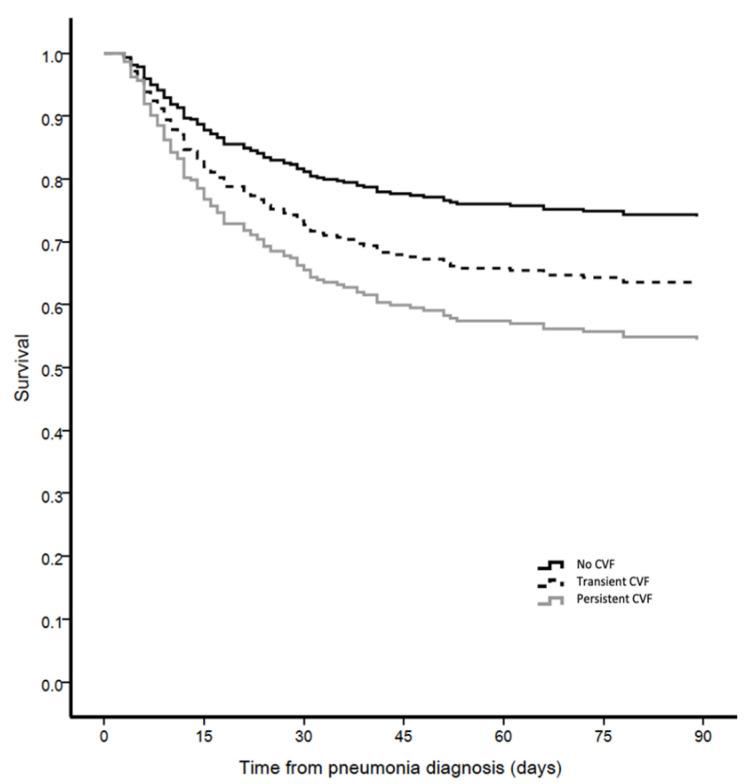
Adjusted survival curve for 90-day mortality in accordance with the presence of cardiovascular failure in patients with ICUAP.

**Figure 2:**
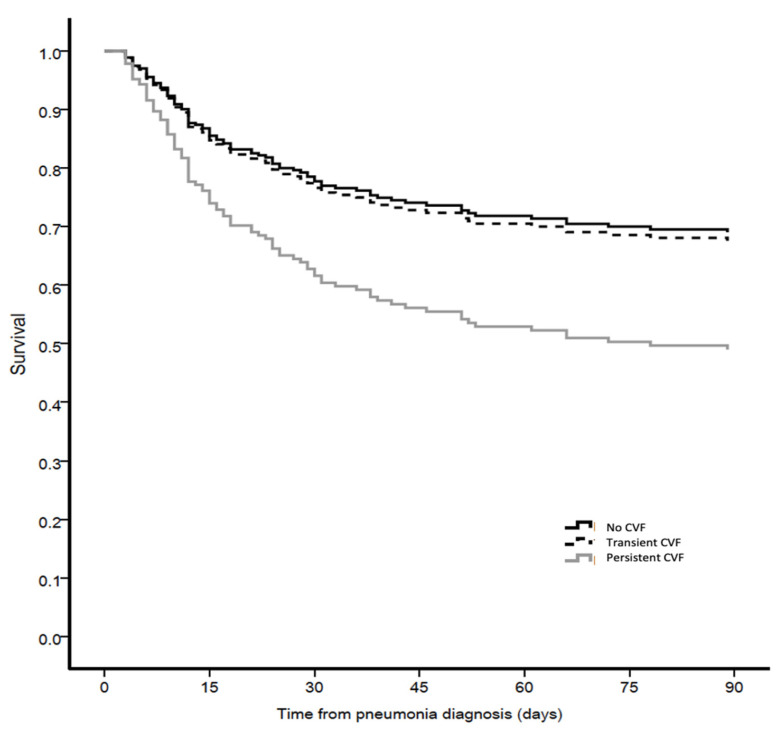
Adjusted survival curve for 90-day mortality in accordance with the presence of cardiovascular failure in patients with microbiologically confirmed ICUAP.

Regarding the primary outcome, as shown in Table 7 and in Figure 1, 90-day mortality in the entire population was higher in patients with persistent CVF (Adjusted HR 2.02, 95% CI 1.29–3.17). This trend was also reported in patients with microbiologically confirmed ICUAP, as shown in Figure 2 (Adjusted HR 1.93, 95% CI 1.11–3.33). Persistent CVF prolonged ICU stay and increased ICU and 28-day mortality. A multivariate analysis was performed to detect independent risk factors correlating with 90-day mortality (Table 8). This table shows that the variables associated with the major outcomes were chronic pulmonary conditions (HR 2.036, 95% CI 1.319–3.144 *p* = 0.001), lack of Pa0_2_/FiO_2_ improvement at day 3 (HR 2.145, 95% CI 1.391–3.308, *p* = 0.001) and persistent CVF (HR 1.771, 95% CI 1.125–2.787, *p* = 0.014). The regression model shows that superinfection (HR 2.354, 95% CI 1.383–4.007, *p* = 0.002), detected at day 3 was also an independent factor associated with higher 90-day mortality.

## 4. Discussion

We describe the natural longitudinal history of critically ill patients with ICUAP and CVF recorded prospectively. The main findings of this study are the following: (1). CVF was common in patients with ICUAP, affecting almost half of the patients; the rates of transient and persistent CVF were similar. (2). CVF was more common in patients presenting certain comorbid conditions (e.g., chronic liver failure) and less common in surgical patients and correlated with the severity of parenchymal lung damage (e.g., ARDS and bilateral infiltrates). (3). CVF was found to be an independent risk factor for predicting invasive mechanical ventilation in patients with ICUAP. (4). Increased levels of inflammation biomarkers were associated with a higher proportion of patients with ICUAP developing CVF; and (5). Patients with CVF had worse prognosis; certain risk factors were also associated with these outcomes, such as chronic pulmonary conditions, lack of oxygenation improvement and persistence of CVF after three days of treatment. (6). The presence of superinfection was also associated with a higher 90-day mortality.

We aimed to analyse the impact of CVF in patients with ICUAP. Previous studies showed that the presence of CVF in patients with VAP was an independent risk factor for poor outcomes. The presence of CVF has been continuously identified as a predictor of mortality in adult patients with VAP. An important addition to the literature that the present study provides is the distinction between transient and persistent CVF. Persistent CVF was an independent risk factor for 90-day mortality and, as noted above, for treatment failure. The distinction between these two CVF entities is clinically relevant, as the prognosis will ultimately be different. We also found that only patients with persistent CVF presented high inflammatory biomarker levels. Few studies have conducted repeated measurements of multiple biomarkers to determine the clinical progression of patients with VAP. A few years ago, in the bioVAP study which assessed the kinetics of CRP, PCT and mid-region fragment of pro-adrenomedullin (MR-proADM), Povoa et al. found that only CRP performed well and correlated with patient survival. More recently, Van Oort et al. analysed the levels of suPAR measured three days before the diagnosis of VAP and found that this biomarker predicted mortality with moderate accuracy. In our cohort, patients with CVF, regardless of the duration of the vasopressor administration, had higher levels of PCT, IL-6, pro-adrenomedullin and SuPAR. These biomarkers might be useful in addition to the methods currently used to diagnose VAP in order to determine the need for vasopressor support.

An interesting and surprising finding was the lack of association between aetiology and the development of CVF. One would have expected certain pathogens to expose the patient to a higher degree of inflammation and more CVF, but only inappropriate antibiotic use was found to produce this exposure. In a multicentre study, Martin-Loeches et al. reported this effect in patients with ventilator-associated lower respiratory tract infections (VA-LRTI) and found that appropriate treatment provided protection only in patients without CVF. In the present study, we also found that patients with persistent CVF more often received inadequate antibiotic treatment likely to lead to treatment failure.

The extent of CVF was not the same in all patients and we found that some subsets of patients were more likely to require vasopressor therapy when developing ICUAP. Among them, multiple trauma patients developed CVF less often than medical patients. Magret et al. reported similar findings in a multicentre European study of patients with VAP, observing that VAP episodes were associated with lower mortality in trauma than in non-trauma patients. However, we also stress that CVF occurred more often in patients with a higher degree of severity both at ICU admission and at onset of pneumonia. This finding was already reported a decade ago, and the usefulness of severity scores, especially APACHE, for prognosis in patients with VAP has since been confirmed.

An important strength of this study is the inclusion not just of patients with VAP but also of non-mechanically ventilated patients with pneumonia. Another interesting finding was that, among patients with non-ventilator ICUAP, those with CVF required subsequent intubation more frequently than those without. This is in line with the recent discussions about the mortality rate of non-ventilated patients requiring invasive mechanical ventilation in the ICU, which is even higher than in patients with VAP, and raises the question of whether patients with CVF require earlier scheduled intubation. In recent years we have seen more patients with non-invasive mechanical ventilation devices in the ICU, but there is also evidence of higher mortality with longer ventilation times. Prospective studies should now be conducted to establish whether the need for vasopressors could be interpreted as a signal of further clinical deterioration.

Our study has certain limitations that should be acknowledged. We analysed the effect of CVF and not CVF because lactate was not available for the current analysis. Previous studies have shown that almost half of patients with vasopressor-dependent CVF do not express lactate on presentation, although this population presents a high mortality rate. While the Sepsis 3 definition is widely used, we consider that the need for vasopressors represents an intermediate stage between sepsis and septic shock; in addition to its advantages for research, in the clinical setting the inclusion of this variable may provide further insights for treatment implementation. Finally, our study was conducted at only one hospital but with a wide range of patients with different baseline and comorbid conditions. Therefore, our findings need to be confirmed in multicentre cohorts.

## 5. Conclusions

In this study, almost half of the patients with ICUAP presented CVF. Patients with CVF had a worse outcome. Only persistent CVF was an independent risk factor for 90-day mortality, along with chronic pulmonary conditions, lack of oxygenation improvement, and superinfection at day 3.

## Figures and Tables

**Table 1:** Baseline characteristics and co-morbidities.

Characteristic	No CVF (*n* = 203)	Transient CVF (*n* = 82)	Persistent CVF (*n* = 73)	*p*-Value	Post-Hoc Comparisons
Age (year)	63.5 ± 16	62.9 ± 14	63.2 ± 13	0.95	
Sex (female)	53 (26%)	24 (29%)	29 (40%)	0.091	
Smoker (current or past)	113 (56%)	37 (45%)	35 (48%)	0.21	
Alcohol abuse (current or past)	57 (28%)	22 (27%)	15 (21%)	0.45	
**Co-morbid conditions**					
Chronic heart disease	73 (36%)	20 (24%)	19 (26%)	0.090	
Chronic lung disease	70 (35%)	24 (29%)	21 (29%)	0.55	
Solid cancer	41 (20%)	13 (16%)	11 (15%)	0.51	
Diabetes	48 (24%)	17 (21%)	17 (23%)	0.87	
Chronic liver disease	28 (14%)	23 (28%)	21 (29%)	0.003	a
Chronic renal failure	14 (7%)	11 (13%)	7 (10%)	0.21	
Chronic systemic steroid use	26 (14%)	8 (11%)	11 (15%)	0.72	
Recent surgery	115 (57%)	35 (43%)	26 (36%)	0.004	b
Previous hospitalization	53 (26%)	24 (29%)	27 (37%)	0.22	
**Severity at ICU admission**					
APACHE-II at ICU admission	15.8 ± 6	18.2 ± 6	18.3 ± 6	0.001	a,b
SOFA at ICU admission				<0.001	a,b
mean	6.6 ± 3	8.3 ± 3	8.4 ± 3		
Median [IQR]	6 [4–9]	8 [6–10]	8 [7–10]		
**Reason for ICU admission**					0.019
Hypercapnic respiratory failure	25 (12%)	10 (12%)	7 (10%)	0.82	
Hypoxemic respiratory failure	30 (15%)	10 (12%)	16 (22%)	0.22	
CVF	15 (7%)	17 (21%)	9 (12%)	0.006	a
Acute coronary syndrome	11 (5%)	4 (5%)	1 (1%)	0.35	
Multiple trauma	22(11%)	2 (2%)	3 (4%)	0.024	
Postoperative	46 (23%)	12 (15%)	12 (16%)	0.22	
Cardiac arrest	7 (3%)	4 (5%)	7 (10%)	0.12	
Decreased consciousness	29 (14%)	8 (10%)	12 (17%)	0.45	
Non-surgical abdominal condition	11 (5%)	6 (7%)	4 (6%)	0.82	
Cardiogenic/hypovolemic shock	4 (2%)	4 (5%)	1 (1%)	0.29	
Other	3 (2%)	5 (6%)	1 (1%)	0.062	
CVF (Septic, Cardio, Hypo)	19 (9%)	21 (26%)	10 (14%)	0.002	a

Abbreviations: SOFA (Sequential Organ Failure Assessment), APACHE II (Acute Physiology and Chronic Health Evaluation II); a: *p* < 0.05 for comparison between the no CVF group and the transient CVF group; b: *p* < 0.05 for comparison between the no CVF group and the persistent CVF group; Alcohol dependence was identified based on diagnose codes in the discharge record.

**Table 2:** Clinical conditions on diagnosis of pneumonia.

Laboratory Parameters	No CVF (n = 203)	Transient CVF (n = 82)	Persistent CVF (n = 73)	*p*-Value	Post-Hoc Comparisons
Temperature	37.0 ± 1.3	36.6 ± 1.5	36.3 ± 1.6	<0.001	b
Leukocytes	13.64 ± 6.43	15.37 ± 7.80	15.20 ± 7.46	0.087	
PaO_2_/FiO_2_	201 ± 80	180 ± 80	184 ± 77	0.076	
Bilateral infiltrates	45 (22%)	28 (34%)	34 (47%)	<0.001	b
Multilobar infiltrates	77 (38%)	41 (51%)	44 (60%)	0.003	b
Pleural effusion	60 (30%)	27 (33%)	32 (45%)	0.074	
ARDS criteria	14 (7%)	13 (16%)	22 (30%)	<0.001	b
CPIS at onset of ICUAP	6.4 ± 1.5	6.7 ± 1.6	7.0 ± 1.6	0.019	b
Corticosteroids at diagnosis	86 (42%)	30 (37%)	30 (41%)	0.67	
**Intubation**					
VAP	118 (58%)	52 (63%)	47 (64%)	0.54	
NV-ICUAP	85 (42%)	30 (37%)	26 (36%)		
Need for intubation among NV-ICUAP	35/85 (41%)	23/30 (77%)	23/26 (89%)	<0.001	a,b
**Severity Scores**					
APACHE II score at ICUAP	14.9 ± 5	18.0 ± 5	18.0 ± 5	<0.001	a,b
SOFA score at ICUAP				<0.001	a,b
mean	5.6 ± 3	10.2 ± 3	10.3 ± 3		
Median [IQR]	5 [4–7]	10 [8–12]	9 [8–12]		
SOFA Score at ICUAP (no cardiovascular)				<0.001	a,b
mean	5.0 ± 2	6.5 ± 3	6.6 ± 3		
Median [IQR]	5 [4–6]	6 [5–8]	6 [5–8]		

Abbreviations: ARDS (Acute Respiratory Distress Syndrome), CPIS (Clinical Pulmonary Infection Score), VAP (Ventilator Associated Pneumonia), SOFA (Sequential Organ Failure Assessment), APACHE II (Acute Physiology and Chronic Health Evaluation II). a: *p* < 0.05 for comparison between the no CVF group and the transient CVF group. b: *p* < 0.05 for comparison between the no CVF group and the persistent CVF group.

**Table 3:** Inflammatory response at pneumonia diagnosis.

Biomarkers	No CVF (n = 203)	Transient CVF (n = 82)	Persistent CVF (n = 73)	*p*-Value	Post-Hoc Comparisons
**CRP** (n = 340)				0.087	
Mean ± SD	13.6 ± 10	15.8 ± 11	16.5 ± 11		
Median [IQR]	12.5 [5.0–19.8]	14.5 [6.5–24.4]	15.0 [8.0–26.2]		
**PCT** (n = 189)				<0.001	a,b
Mean ± SD	0.75 ± 1	2.9 ± 6	5.1 ± 15		
Median [IQR]	0.24 [0.11–0.87]	0.72 [0.36–3.29]	0.73 [0.16–2.44]		
**IL-6** (n = 185)				<0.001	a,b
Mean ± SD	219 ± 433	722 ± 1155	570 ± 868		
Median [IQR]	94 [39–194]	240 [57–739]	216 [109–511]		
**IL-8** (n = 185)				0.070	
Mean ± SD	540 ± 3226	419 ± 1322	842 ± 3424		
Median [IQR]	89 [52–149]	99 [66–214]	114 [73–283]		
**TNF-alpha** (n = 185)				0.21	
Mean ± SD	10.6 ± 12	16.1 ± 31	14.0 ± 17		
Median [IQR]	7 [5–11]	9 [5–18]	9 [5–17]		
**Pro-adrenomedullin** (n = 205)				0.004	a
Mean ± SD	1.54 ± 2	3.27 ± 4	3.03 ± 4		
Median [IQR]	1.10 [0.33–1.91]	1.71 [0.94–3.50]	1.36 [0.75–4.04]		
**suPAR** (n = 176)				0.012	b
Mean ± SD	6.93 ± 5	10.67 ± 9	10.76 ± 8		
Median [IQR]	5.5 [3.6–9.0]	6.9 [4.4–15.5]	10.0 [4.8–15.5]		

Abbreviations: CRP (C-reactive protein), PCT (procalcitonin), IL-6 (interleukin-6), IL-8 (interleukin 8), TNF-alpha (Tumor necrosis factor alpha), suPAR (soluble urokinase-type plasminogen activator receptor). a: *p* < 0.05 for comparison between the no CVF group and the transient CVF group. b: *p* < 0.05 for comparison between the no CVF group and the persistent CVF group.

**Table 4:** Inflammatory response at Day 3.

Biomarkers	No CVF (n = 203)	Transient CVF (n = 82)	Persistent CVF (n = 73)	*p*-Value	Post-Hoc Comparisons
**CRP** (n = 320)				0.002	b
Mean ± SD	10.7 ± 8	12.2 ± 10	16.4 ± 11		
Median [IQR]	9.18 [4.5–15.8]	8.6 [4.0–17.6]	16.7 [5.3–25.9]		
**PCT** (n = 163)				0.001	a,b
Mean ± SD	0.93 ± 4	2.2 ± 5	3.0 ± 10		
Median [IQR]	0.16 [0.09–0.47]	0.64 [0.14–2.21]	0.58 [0.15–1.80]		
**IL-6** (n = 155)				0.001	b
Mean ± SD	137 ± 319	208 ± 333	325 ± 552		
Median [IQR]	66 [15–141]	100 [41–224]	163 [52–306]		
**IL-8** (n = 155)				0.63	
Mean ± SD	675 ± 3029	472 ± 2117	128 ± 102		
Median [IQR]	71 [43–137]	80 [41–145]	82 [59–160]		
**TNF-alpha** (n = 155)				0.219	
Mean ± SD	11.5 ± 22	9.7 ± 7	11.2 ± 7		
Median [IQR]	7 [5–10]	7 [5–13]	11 [5–15]		
**Pro-adrenomedullin** (n = 179)				0.003	b
Mean ± SD	1.52 ± 2	2.36 ± 3	2.92 ± 3		
Median [IQR]	0.94 [0.35–1.75]	1.51 [0.58–2.67]	1.73 [0.91–3.26]		
**SuPAR** (n = 148)				0.022	b
Mean ± SD	7.16 ± 5	9.95 ± 7	12.01 b ± 9		
Median [IQR]	6.2 [4.2–8.9]	7.5 [4.5–13.3]	10.6 [4.4–16.0]		

Abbreviations: CRP (C-reactive protein), PCT (procalcitonin), IL-6 (interleukin-6), IL-8 (interleukin 8), TNF-alpha (Tumor necrosis factor alpha), suPAR (soluble urokinase-type plasminogen activator receptor). a: *p* < 0.05 for comparison between the no CVF group and the transient CVF group. b: *p* < 0.05 for comparison between the no CVF group and the persistent CVF group.

**Table 5:** Aetiology and antibiotic treatment.

Aetiology and Antibiotic Treatment	No CVF (n = 203)	Transient CVF (n = 82)	Persistent CVF (n = 73)	*p*-Value	Post-Hoc Comparisons
Defined causative pathogen	120 (59%)	56 (68%)	52 (71%)	0.11	
Multiple causative pathogens (polymicrobial)	29 (14%)	12 (15%)	16 (22%)	0.29	
Multi-drug resistant pathogen	28/120 (23%)	16/56 (29%)	12/52 (23%)	0.72	
Empiric antibiotic treatment according to ATS guidelines	133 (67%)	51 (65%)	49 (68%)	0.89	
Inadequate initial antibiotic treatment	18/120 (15%)	7/56 (13%)	18/52 (35%)	0.004	b,c
Treatment failure	91 (45%)	44 (54%)	57 (78%)	<0.001	b,c
Antibiotic change	134 (66%)	46 (56%)	52 (71%)	0.12	
Superinfection at day 3	11 (5%)	9 (11%)	13 (18%)	0.006	b

b: *p* < 0.05 for comparison between the no CVF group and the persistent CVF group. c: *p* < 0.05 for comparison between the transient CVF group and the persistent CVF group.

**Table 6:** Causative pathogens.

Causative Pathogens	No CVF (n = 120)	Early Transient CVF (*n* = 56)	Early Persistent CVF (*n* = 52)	*p*-Value	Post-Hoc Comparisons
Gram negative non-fermenting bacteria	41 (34%)	23 (41%)	25 (48%)	0.21	
*P. aeruginosa*	36 (30%)	20 (36%)	22 (42%)	0.28	
*S. aureus*	38 (32%)	12 (21%)	14 (27%)	0.36	
MSSA	26 (22%)	7 (13%)	11 (21%)	0.33	
MRSA	12 (10%)	5 (9%)	3 (6%)	0.67	
Gram negative enteric bacteria	37 (31%)	17 (30%)	14 (27%)	0.87	
Community-acquired pathogens (*S. pneumoniae*, *H. influenza*, etc.)	12 (10%)	7 (13%)	4 (8%)	0.71	
*Aspergillus spp.*	7 (6%)	1 (2%)	4 (7%)	0.36	
Other	8 (7%)	5 (9%)	4 (8%)	0.87	

Abbreviations: *P. aeruginosa* (*Pseudomonas aeruginosa*), *S. aureus* (*Staphylococcus aureus*), MSSA (Methicillin-Sensitive *Staphylococcus aureus*), MRSA (methicillin-resistant *Staphylococcus aureus*), *S. pneumoniae* (*Streptococcus pneumoniae*), *H. influenza* (*Haemophilus influenzae*).

**Table 7:** Primary and secondary outcomes.

Primary and Secondary Outcomes	No CVF (n = 120)	Transient CVF (n = 56)	Persistent CVF (n = 52)	*p*-Value	Post-Hoc Comparisons
90-day mortality n (%)	62 (31%)	39 (48%)	45 (62%)	<0.001	b
ICU stay, days				0.005	b,c
Mean ± SD	19.6 ± 18	18.6 ± 14	29.0 ± 26		
Median [IQR]	14 [8–24]	14 [9–22]	24 [10–40]		
Hospital stay, days					a,c
Mean ± SD	44.9 ± 37	35.8 ± 23	49.3 ± 43	0.17	
Median [IQR]	38 [20–56]	29 [19–46]	39 [19–64]		
28-day mortality, n (%)	39 (19%)	29 (35%)	30 (41%)	<0.001	b
ICU mortality, n (%)	38 (19%)	31 (38%)	44 (60%)	<0.001	a,b,c

Abbreviations: ICU (Intensive Care Unit), IQR (Interquartile range), SD (Standard deviation). a: *p* < 0.05 for comparison between the no CVF group and the transient CVF group. b: *p* < 0.05 for comparison between the no CVF group and the persistent CVF group. c: *p* < 0.05 for comparison between the transient CVF group and the persistent CVF group.

**Table 8:** Multivariate Cox regression for 90-day mortality.

**(a)**			
Exposure *	**Category**	**Adjusted HR** (95% CI) *	***p*-Value**
			**0.008**
No CVF	Reference	1	
Transient CVF	Yes/No	1.53 (0.95–2.46)	0.082
Persistent CVF	Yes/No	2.02 (1.29–3.17)	0.002
**(b)**			
Exposure *	**Category**	**Adjusted HR** (95% CI) *	***p*-Value**
			**0.044**
No CVF	Reference	1	
Transient CVF	Yes/No	1.06 (0.59–1.91)	0.85
Persistent CVF	Yes/No	1.93 (1.11–3.33)	0.019

* Adjusted for age, VAP/NV-ICUAP, all chronic comorbidities, non-cardiovascular total SOFA at pneumonia diagnosis, APACHE II at pneumonia diagnosis, multilobar infiltrates, recent surgery, ICU admission reason, corticosteroid use at pneumonia diagnosis, microbiology defined, ATS guidelines, empiric adherence and adequate empirical antibiotic treatment. CVF: Cardiovascular failure.

## Data Availability

The anonymized database collected for the study by the Hospital Clinic, and the data dictionary that defines each field in the set, will be made available to reviewers if they consider it necessary prior confidentiality agreement.
